# Metabolic-immune axis in the tumor microenvironment: a new strategy for prognostic assessment and precision therapy in DLBCL and FL

**DOI:** 10.3389/fimmu.2025.1659011

**Published:** 2025-10-01

**Authors:** Chengqian Chen, Wei Guo, Haotian Wang, Luming Cao, Ou Bai

**Affiliations:** Department of Hematology,The First Hospital of Jilin University, Changchun, China

**Keywords:** tumor metabolism, immune microenvironment, metabolic-immune axis, lymphoma, targeted therapy

## Abstract

Diffuse large B-cell lymphoma and follicular lymphoma exhibit complex metabolic and immune microenvironments that influence disease progression and treatment response. Metabolic reprogramming, including glycolysis, amino acid, and lipid metabolism, supports tumor growth while suppressing anti-tumor immunity. Immune components such as tumor-infiltrating lymphocytes and checkpoint molecules (PD-L1, LAG-3, TIM-3) further modulate prognosis. Elevated tumor metabolic volume and glycolytic activity correlate with aggressive disease and poor outcomes. Conversely, high TIL density often predicts better responses. Integrating metabolic and immune biomarkers enhances risk stratification and therapeutic strategies, highlighting the potential for combined metabolic inhibitors and immunotherapies to improve precision medicine in lymphoma.

## 1 Introduction

Diffuse Large B-Cell Lymphoma (DLBCL) and Follicular Lymphoma (FL) are the two most common subtypes of non-Hodgkin lymphoma (NHL) (1–3). Despite significant differences in their biological behavior, clinical features, and treatment strategies, both exhibit high heterogeneity and complex tumor microenvironments (TME) (1–3). From a cellular origin perspective, DLBCL primarily arises from germinal center B cells or activated B cells, with tumor cells typically extensively involving lymph nodes and extranodal organs (e.g., gastrointestinal tract, central nervous system) (4, 5). FL originates from germinal center B cells, with tumor cells proliferating mainly in lymph nodes and the spleen to form follicular structures (6, 7).

Currently, the standard first-line treatment for DLBCL is the R-CHOP regimen (combination chemotherapy with rituximab, cyclophosphamide, doxorubicin, vincristine, and prednisone) (8, 9). Treatment strategies for FL are more diverse, determined by disease grading and staging, and include watchful waiting, rituximab monotherapy or in combination with chemotherapy, as well as emerging immunomodulators and targeted therapies (10, 11). Although R-CHOP achieves cure in some DLBCL patients, 30–40% still face recurrence or refractory disease (12, 13). FL poses long-term management challenges due to its high recurrence rate and risk of transforming into more aggressive DLBCL (14, 15). The heterogeneity of these diseases and the diversity of treatment responses suggest that, beyond traditional clinical and pathological features, metabolic and immune factors within the TME may play a critical role in disease progression and prognosis.

Recent studies on tumor metabolic reprogramming and the immune microenvironment have provided new insights into the biological behavior of DLBCL and FL (16). Through metabolic reprogramming, tumor cells not only meet their rapid proliferation energy demands but may also reshape the immune microenvironment via metabolic byproducts, thereby suppressing antitumor immune responses (17, 18). For instance, Tumor Metabolic Volume (TMV) and glycolytic activity have been demonstrated to correlate with disease aggressiveness and prognosis in both DLBCL and FL (19–21). Concurrently, tumor-infiltrating lymphocytes (TILs), programmed death-ligand 1 (PD-L1) expression levels, and the distribution of immune checkpoint molecules within the immune microenvironment also play crucial roles in regulating antitumor immune responses (22, 23). High TIL density is typically associated with favorable treatment response and prognosis (24), whereas infiltration of immunosuppressive cells (e.g., regulatory T cells (Tregs), M2 macrophages) may promote immune escape and disease progression (25, 26). Given the critical importance of tumor-microenvironment interactions, therapeutic strategies targeting these crosstalk pathways—such as using immune checkpoint inhibitors to reverse T cell exhaustion or employing BTK inhibitors to modulate BCR signaling and the microenvironment—have emerged as novel research focuses.

Therefore, exploring the roles of metabolic parameters (e.g., tumor microenvironment volume, glycolytic activity) and immune parameters (e.g., TIL density, PD-L1 expression) in DLBCL and FL not only helps elucidate the biological mechanisms of the disease but may also provide novel biomarkers and therapeutic targets for risk stratification, prognostic prediction, and personalized treatment.

This review aims to systematically summarize research advances on the metabolic and immune microenvironments in DLBCL and FL, explore their relationship with disease risk and prognosis, and discuss their potential applications in clinical translation. By integrating metabolic and immune parameters, we hope to provide novel insights for improving treatment strategies in DLBCL and FL, thereby advancing precision medicine in this field.

## 2 Interplay between metabolism and immunity

**Figure 1:**
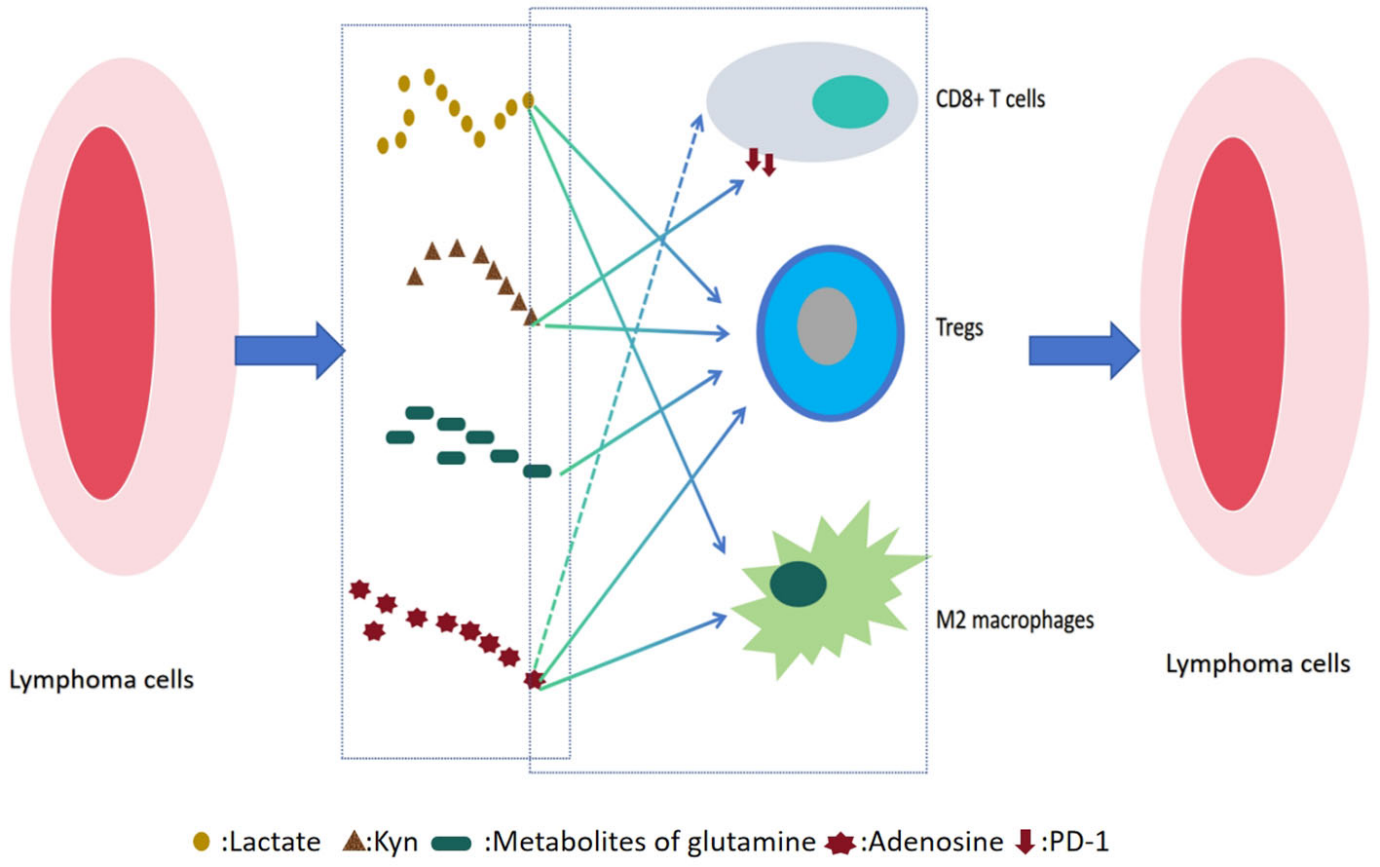
Metabolic immune interaction network in lymphoma microenvironment.

In DLBCL and FL, the metabolic reprogramming of the TME forms a dynamic and reciprocal regulatory network with immune cell function, playing a pivotal role in lymphoma progression(as shown in **Figure 1**). Tumor cells enhance glycolysis and amino acid metabolism not only to fuel rapid proliferation and biosynthesis but also to reshape the local microenvironment through the release of metabolic byproducts such as lactate, glutamine, and adenosine. These metabolites can directly suppress the cytotoxic functions of effector immune cells such as CD8+ T cells and natural killer (NK) cells, while promoting the expansion of immunosuppressive populations like Tregs and M2-polarized macrophages.

Importantly, immune cells are not merely passive responders. Activated T cells can secrete interferon-gamma (IFN-γ) to disrupt tumor metabolic pathways, and the polarization state of macrophages (M1 versus M2) can in turn modulate tumor glycolysis and mitochondrial metabolism. This bidirectional metabolic–immune crosstalk profoundly influences lymphoma heterogeneity, therapeutic resistance, and patient prognosis, providing a strong rationale for the development of combined metabolic and immune-targeted treatment strategies.

### 2.1 Effects of metabolic reprogramming on immune cells

Metabolic reprogramming in tumor cells has emerged as a central hallmark in cancer biology, playing a pivotal role in tumor initiation and progression. Recent advances in TME research have highlighted that metabolic alterations—particularly enhanced glycolysis and amino acid metabolism—not only fulfill the energetic and biosynthetic demands of rapidly proliferating tumor cells but also profoundly reshape the immune milieu, suppressing anti-tumor immune responses and facilitating immune evasion and disease progression.

#### 2.1.1 Immunomodulatory effects of glycolysis and lactate metabolism

Glycolysis and lactate accumulation play central roles in tumor-associated immune suppression. Tumor cells exhibit a distinctive metabolic phenotype known as the Warburg effect, in which they preferentially utilize glycolysis for energy production even under normoxic conditions. This leads to substantial lactate accumulation and acidification of the TME (27). Lactate is extruded from tumor cells through monocarboxylate transporters (MCTs, primarily MCT4) (28–30). Subsequently, it enters the tumor microenvironment via MCT1, which is mainly expressed on immune cells, thus decreasing the extracellular pH value (28–30). Notably, lactate dehydrogenase (LDH), a key enzyme in lactate metabolism, is markedly overexpressed in DLBCL and other tumors. The lactate produced by LDH impairs the cytotoxic functions of CD8+ T cells and NK cells, thereby weakening the host’s anti-tumor immunity (31–33). In addition, lactate promotes the expansion of Tregs and the polarization of macrophages toward an M2 immunosuppressive phenotype (34–37). Moreover, lactate inhibits the differentiation and antigen-presenting capacity of dendritic cells, further disrupting T cell activation and facilitating immune escape (31, 37, 38). According to the Warburg effect, lactic acidosis can occur when lactate homeostasis is disrupted due to overproduction and/or reduced utilization. Lactic acidosis is classified into two types: type A, caused by tissue hypoxia, and type B, which occurs under normoxic conditions due to non-hypoxic factors such as drugs or toxins. Type B lactic acidosis is associated with altered glycolysis and redox imbalance (39). It is frequently observed in human malignancies—particularly lymphomas—and is associated with poor prognosis if not treated promptly (40–42). Another significant cause of lactic acidosis is thiamine deficiency, which is a distinct feature associated with type B lactic acidosis (43, 44). This mechanism has important clinical significance, as thiamine is a key coenzyme in the pyruvate dehydrogenase complex, responsible for converting the glycolytic end product pyruvate into acetyl CoA, which enters the tricarboxylic acid cycle (43). When thiamine is deficient, pyruvate cannot be oxidized normally, resulting in a large amount of conversion to lactate under the action of LDH, leading to severe type B lactic acidosis (43). In lymphoma patients, thiamine deficiency may be caused by high tumor consumption, loss of appetite, or treatment side effects (45). Identifying and correcting this condition is crucial for managing such tumor emergencies and improving patient prognosis (45). Clinically, elevated serum LDH levels are significantly associated with poor prognosis in DLBCL and have been incorporated into the International Prognostic Index (IPI). Similarly, LDH has prognostic value in FL and is a component of the Follicular Lymphoma International Prognostic Index (FLIPI). As a direct marker of tumor metabolic activity, elevated LDH reflects increased tumor burden and the formation of a lactate-mediated immunosuppressive microenvironment.

#### 2.1.2 Immunoregulatory effects of amino acid metabolism

Abnormal amino acid metabolism is another crucial mechanism underlying tumor immune evasion. In tryptophan metabolism, CD11c+ myeloid dendritic cells (mDCs) within the FL microenvironment exhibit high expression of indoleamine 2,3-dioxygenase 1 (IDO1), which depletes tryptophan and leads to the accumulation of kynurenine (Kyn) (46). This process promotes immune tolerance through two mechanisms: first, Kyn activates the aryl hydrocarbon receptor (AhR) pathway, inducing Treg differentiation and suppressing effector T cell function (47, 48); second, Kyn-AhR signaling enhances PD-1 expression on CD8+T cells via intercellular signaling within the TME, resulting in a PD-1+ exhausted T cell phenotype (49). Quantification of Kyn and tryptophan levels has shown promise as a prognostic biomarker in both DLBCL and FL (46, 50, 51). Furthermore, inhibitors targeting key enzymes in tryptophan metabolism (e.g.,IDO1/TDO) have demonstrated therapeutic potential in preclinical models, providing a new avenue for precision therapy in lymphoma (52–54). Serine metabolism also supports tumor growth, survival, and adaptation to hostile microenvironments through multiple pathways and has emerged as a promising therapeutic target (55). The rate-limiting enzyme in the serine synthesis pathway (SSP), phosphoglycerate dehydrogenase (PHGDH), is frequently overexpressed or amplified in various cancers, making it a critical vulnerability for therapeutic intervention (56). In DLBCL, PHGDH overexpression is strongly associated with MYC activation, particularly in the germinal center B-cell-like (GCB) subtype. This may drive chemotherapy resistance through enhanced serine flux, reducing the efficacy of R-CHOP and shortening overall survival (57). In addition, serine metabolic enzymes interact with immunosuppressive cells in the TME—such as M2 macrophages—potentially modulating responses to immunotherapies like PD-1 inhibitors (58, 59). Future studies should investigate the therapeutic potential of PHGDH inhibitors in lymphoma, with the aim of overcoming resistance and improving efficacy through combination strategies. It is also essential to identify predictive biomarkers to select patient subgroups most likely to benefit from such approaches.

#### 2.1.3 Immunomodulatory role of glutamine metabolism

As a central node in tumor energy metabolism, glutamine metabolism plays a critical role in both DLBCL and FL. This pathway begins with the active uptake of glutamine via transporters such as ASCT2/SLC1A5, followed by its conversion into glutamate by glutaminase (GLS), and subsequently into α-ketoglutarate (α-KG), which enters the tricarboxylic acid (TCA) cycle to provide energy and biosynthetic precursors for tumor cells (60, 61). Notably, glutamine metabolism also modulates the tumor immune microenvironment through multiple mechanisms: it competes with effector T cells for nutrients, thereby limiting their metabolic fitness (62), supports the survival and function of immunosuppressive cells such as Tregs and myeloid-derived suppressor cells (MDSCs) (63), and is involved in the regulation of immune checkpoint molecules such as PD-L1 (64). Metabolomic profiling reveals elevated levels of glutamine catabolites in the plasma of lymphoma patients (65), and all tested DLBCL cell lines express GLS1 regardless of subtype classification (66). GLS1 inhibition using CB-839, in combination with the BCL2 inhibitor ABT-199, not only induces significant reactive oxygen species (ROS) production but also exerts synergistic cytotoxicity—suggesting that co-targeting GLS1 and BCL2 could be a promising therapeutic strategy in DLBCL (66). Furthermore, recent studies have uncovered a key mechanism of glutamine metabolic reprogramming in DLBCL involving the mitochondrial pyruvate carrier (MPC) and glutamate-pyruvate transaminase 2 (GPT2) pathway (67). Studies have found that DLBCL can be further classified according to cell origin and molecular characteristics. OXPHOS DLBCL (oxidative phosphorylation subtype) is mainly manifested in the activation of oxidative phosphorylation metabolic pathway and the enhancement of mitochondrial function, while BCR-DLBCL (B cell receptor subtype) is highly dependent on B cell receptor signaling pathway and its downstream glycolysis process (68, 69). Because of this difference in metabolic dependence, the sensitivity of the two subtypes to targeted treatment strategies is also different: OXPHOS DLBCL may be more sensitive to inhibitors targeting oxidative phosphorylation related pathways such as glutamine metabolism, while BCR-DLBCL may be more responsive to drugs targeting BCR signaling pathways such as Btk inhibitors. It is worth noting that although these two subtypes exhibit distinct metabolic characteristics, they are highly dependent on glutamine metabolism to maintain the activity of the TCA cycle (66, 70). GPT2-mediated α-KG production requires mitochondrial pyruvate input, which is dependent on MPC activity. Under extracellular matrix (ECM)-mimicking conditions that better simulate solid tumor environments, MPC inhibition significantly reduces α-KG production and suppresses DLBCL proliferation—an effect not observed in conventional suspension culture, highlighting the critical influence of the microenvironment on metabolic behavior (67). Moreover, MPC inhibition increases DLBCL sensitivity to ammonia, due to impaired ammonia detoxification via glutamate dehydrogenase (GDH) under ECM conditions (67). These findings challenge traditional views of DLBCL metabolism and suggest therapeutic potential for targeting the MPC–GPT2 axis. Future studies should investigate the feasibility of GPT2-specific inhibitors and explore how microenvironment-driven metabolic changes influence treatment resistance.

#### 2.1.4 Immunomodulatory role of lipid metabolism

Lipid metabolism plays a dual role in the development of DLBCL and FL: on one hand, it directly supports tumor cell proliferation and survival, and on the other, it shapes the tumor immune microenvironment to facilitate immune escape. In B-cell malignancies, constitutive activation of the BCR-PI3K-AKT signaling pathway leads to mTORC1 hyperactivation, which upregulates anabolic processes including lipid and cholesterol biosynthesis (71). Cholesterol synthesis driven by this pathway may form a positive feedback loop that sustains BCR signaling, further promoting lymphoma progression. Cholesterol, a key component of membrane homeostasis, is essential for tumor cell proliferation. Lymphoma cells utilize cholesterol via BCR signaling to maintain proliferative and pro-survival pathways. Excess free cholesterol is rapidly esterified by Acetyl coenzyme A acetyltransferase (ACAT) or exported via transporters such as scavenger receptor class B type I (SR-BI) and ATP-binding cassette protein A1—processes particularly evident in pathological conditions like macrophage foam cell formation (72). Targeting cholesterol metabolism has emerged as a novel therapeutic strategy in lymphoma. SR-BI inhibitors have shown anti-lymphoma activity by disrupting cholesterol homeostasis (72), and metformin has been reported to improve prognosis in DLBCL patients with type 2 diabetes, potentially by modulating cholesterol metabolism (73). Additionally, lipid metabolism-related gene signatures have been validated as independent prognostic factors in DLBCL, enhancing predictive accuracy when combined with the IPI (74, 75). Clinical observations suggest that statins, when used alongside standard chemoimmunotherapy in the rituximab era, do not compromise treatment efficacy in DLBCL/FL, although their potential benefit in FL remains to be further confirmed (76). Within the TME, lipids represent a double-edged sword—they can both support and suppress anti-tumor immunity. For instance, enhanced fatty acid oxidation (FAO) promotes the expansion of tumor-reactive CD8+ T cells and improves response to PD-1 blockade (77, 78); Conversely, lipid uptake by CD8+ T cells via CD36 leads to lipid peroxidation and ferroptosis, impairing their effector function and weakening anti-tumor immunity (79, 80). Therefore, metabolic interventions must carefully balance the dynamic demands of both tumor and immune cells. Future strategies should integrate metabolomics with immune profiling to develop precise approaches that overcome the “double-edged sword” nature of metabolic reprogramming, ultimately facilitating more effective clinical translation.

#### 2.1.5 Immunomodulatory role of adenosine metabolism

Adenosine metabolism plays a key immunosuppressive role in the tumor immune microenvironment. The main pathway of extracellular ATP production is the enzymatic cascade reaction of CD39 and CD73 (ATP→ADP→AMP→adenosine), while intracellular AMP can also catalyze the production of adenosine under energy stress (81–84). In DLBCL and FL, high expression of CD39—particularly in the non-GCB subtype of DLBCL—is strongly associated with poor prognosis. This effect is mediated by adenosine binding to A2A receptors (A2AR), triggering a broad spectrum of immunosuppressive responses, including inhibition of CD8+ T and NK cell activity, expansion of Tregs, polarization of macrophages toward the M2 phenotype, and impairment of dendritic cell antigen presentation (85–87). Hypoxic conditions in the TME further induce CD39/CD73 expression via HIF-1α, leading to increased adenosine accumulation. This process synergizes with other immunosuppressive pathways such as lactate metabolism and IDO1-mediated tryptophan catabolism, forming a tightly integrated immunosuppressive network (88–91). Current therapeutic approaches focus on inhibitors targeting CD39/CD73 and A2AR antagonists. Preclinical studies suggest that combining these agents with PD-1 inhibitors can reverse immune suppression and restore anti-tumor responses (92, 93). Future directions include the development of precision stratification methods based on CD39/CD73 expression and investigation of combination strategies targeting the hypoxia–CD39/CD73–A2AR axis to overcome immune evasion in lymphoma.

### 2.2 Immune cell-mediated regulation of tumor metabolism

Within the TME, immune cells and tumor cells engage in a complex and dynamic bidirectional interaction that profoundly influences tumor initiation, progression, and response to therapy. Immune cells are not merely passive victims of tumor metabolic reprogramming; they actively modulate the metabolic status of tumor cells through cytokine secretion and direct cell–cell contact. This reciprocal regulation is of significant theoretical and clinical importance, particularly in the context of lymphomas.

#### 2.2.1 Tumor-infiltrating regulatory T cells and tumor metabolism

Tumor-infiltrating Tregs exert potent immunosuppressive effects in the TME through metabolic mechanisms, thereby promoting immune evasion (94–96). The key Treg transcription factor FOXP3 suppresses glycolysis while enhancing OXPHOS and NAD+ oxidation, enabling Tregs to survive and function under hypoglycemic and hypoxic conditions commonly found in the TME (97, 98). This adaptation relies on activation of the LKB1/AMPK pathway, where LKB1 phosphorylates AMPK to enhance mitochondrial metabolic efficiency while inhibiting mTORC1 signaling—collectively preserving Treg stability under metabolic stress (99, 100). Tregs contribute to immunosuppression not only through secretion of cytokines such as TGF-β, IL-10, and IL-35 but also by competitively depleting key nutrients such as glucose, glutamine, and tryptophan—thereby impairing effector T cell function. Specifically, TGF-β suppresses the cytotoxic activity of NK cells and cytotoxic T lymphocytes (CTLs) and promotes the transdifferentiation of Th17 cells into Tregs, exacerbating immune suppression (101, 102). IL-10 directly inhibits effector T cell cytotoxicity, while IL-35 promotes T cell exhaustion (103, 104). Notably, under nutrient-deprived conditions, Tregs can utilize abundant metabolic byproducts in the TME—such as lactate and fatty acids—to maintain their function (105, 106). Additionally, through the CD39/CD73–adenosine axis and IDO–kynurenine pathway, Tregs secrete immunosuppressive metabolites (e.g., adenosine and kynurenine), which further inhibit effector T cells and reinforce tumor glycolytic reprogramming—forming a self-reinforcing immunosuppressive loop (48, 107, 108). This metabolic advantage correlates with adverse clinical outcomes. High Treg infiltration in DLBCL and FL is associated with poor treatment response, increased risk of disease progression, and shorter progression-free survival (PFS) (109–111). Moreover, LKB1 has been shown to promote DLBCL immune evasion by enhancing Treg metabolic stability and suppressive activity (112). Therefore, targeting Treg-specific metabolic pathways—such as CD39/CD73, IDO, or fatty acid oxidation—represents a promising approach to reversing the immunosuppressive microenvironment and boosting anti-tumor immunity.

#### 2.2.2 Interplay between CD8+ T cells and tumor metabolism

CD8+ T cells are the principal effectors of adaptive anti-tumor immunity (113, 114). Studies have shown that CD8+ TILs secrete IFN-γ, which acts on tumor cells through the JAK–STAT pathway to suppress glycolysis (i.e., reversal of Warburg effect) and mitochondrial oxidative phosphorylation—thereby limiting tumor energy metabolism and biosynthetic capacity (114–117). However, lymphoma cells can evade immune surveillance through multiple mechanisms. IFN-γ induces tumor cells to upregulate PD-L1, which binds to PD-1 on CD8+ T cells, leading to their inactivation, exhaustion, and reduced proliferation—ultimately establishing an immunosuppressive feedback loop (118–120). Additionally, lymphoma cells reprogram metabolism (e.g., increasing lactate production, competitively consuming glucose and amino acids in the TME) to further suppress CD8+ T cell function and facilitate immune evasion (121).

In DLBCL, both the density and functional status of CD8+ TILs are significantly associated with treatment response to R-CHOP (122). High CD8+ T cell infiltration predicts better complete response rates and prolonged PFS, suggesting that CD8+T cell-mediated immunometabolic regulation may influence chemosensitivity (123, 124). A similar relationship has been observed in FL, where CD8+ TILs correlate with lower progression risk and improved immunochemotherapy outcomes (125, 126).

In recent years, immunotherapy for CD8+T cell metabolism has made progress in relapsed/refractory DLBCL and FL, but the problem of drug resistance needs to be solved urgently (127–130). Recent studies have found that the abnormally activated fibroblast activation protein (FAP) - positive fibroblast reticular cells (FRCS) in DLBCL can inhibit the function of CD8+T cells, and the combination of FAP targeted drugs and glofitamab can significantly enhance the anti-tumor activity of TIL (131). This suggests that simultaneously targeting T cell metabolism and tumor microenvironment may be a new direction to overcome drug resistance. FAP is a type II transmembrane serine protease, which is characterized by high expression on the surface of cancer associated fibroblasts (CAF), but is highly restricted in most normal adult tissues. This unique expression pattern makes it a potential therapeutic target in the tumor microenvironment (132–134).

#### 2.2.3 Impact of macrophage polarization on tumor metabolism

Macrophages, as abundant and functionally diverse immune cells within the TME, exert significant influence on tumor metabolism depending on their polarization status (135, 136). Owing to their plasticity, macrophages can differentiate into distinct functional phenotypes in response to environmental cues, primarily the classically activated M1 phenotype and the alternatively activated M2 phenotype (135, 136). These polarization states exhibit opposing roles in tumor metabolic regulation and thus critically shape tumor progression and therapeutic responses (135, 136).

M1 macrophages, considered anti-tumor “guardians,” are typically induced by proinflammatory stimuli such as IFN-γ and lipopolysaccharide (LPS) (137–139). They exert cytotoxic effects through secretion of tumor necrosis factor-α (TNF-α), nitric oxide (NO), and ROS, which not only directly kill tumor cells but also suppress their metabolic activity (140). Functionally, M1 macrophages undergo metabolic reprogramming characterized by a shift from oxidative phosphorylation (OXPHOS) to aerobic glycolysis (Warburg effect), enabling rapid ATP generation and provision of metabolic intermediates to sustain proinflammatory responses (141, 142). High expression of inducible nitric oxide synthase (iNOS) in M1 macrophages promotes arginine catabolism to NO, which damages mitochondrial function and induces apoptosis in tumor cells (143). Conversely, M2 macrophages are induced by anti-inflammatory cytokines such as IL-4, IL-10, and IL-13, and generally exhibit tumor-promoting properties (140). M2 macrophages rely primarily on OXPHOS for energy production, which allows them to adapt to the hypoxic and nutrient-depleted TME (142). This may seem contradictory, but it actually stems from its metabolic flexibility. M2 macrophages maintain energy homeostasis and perform tumor promoting functions under hypoxic conditions by enhancing mitochondrial efficiency, using alternative substrates (such as fatty acids) for oxidative phosphorylation, and coordinating HIF-1 α and AMPK signaling pathways (142, 144). In FL—an indolent B-cell malignancy that may transform into aggressive DLBCL—macrophage polarization is closely linked to tumor metabolism and disease progression (145). A high infiltration of M2 macrophages in FL patients correlates with increased risk of relapse and poorer prognosis (146). Mechanistically, M2 macrophages promote FL progression via multiple pathways. First, cytokines such as IL-10 and TGF-β activate tumor-intrinsic PI3K-AKT-mTOR and STAT3 signaling pathways, enhancing glycolysis and lipid biosynthesis in lymphoma cells (147, 148); Second, M2 macrophages suppress anti-tumor immune responses, fostering an immune-privileged niche for tumor survival (149). Collectively, these effects enhance tumor proliferation, drug resistance, and transformation risk in FL.

In conclusion, the polarization state of macrophages plays a key role in the regulation of tumor metabolism. Intervention strategies for macrophage polarization (such as promoting M1 polarization or inhibiting M2 polarization) may become a new direction to improve tumor microenvironment and enhance anti-tumor efficacy (150–153). Especially in lymphoma such as DLBCL and FL, the treatment method targeting macrophage polarization is expected to provide a new breakthrough for inhibiting disease progression (154–156).

#### 2.2.4 Cytokine-mediated regulation of tumor metabolism

Cytokines serve as pivotal mediators of immune cell communication and play key roles in shaping tumor metabolic phenotypes (157). In both DLBCL and FL, cytokines modulate metabolic reprogramming to promote tumor progression (158). IL-6 and IL-10, for example, activate the STAT3 pathway to enhance glycolysis and glutathione metabolism, supplying energy and biosynthetic precursors for rapid tumor cell proliferation (159, 160). Although IL-16 was previously linked to cutaneous T-cell lymphoma (161), recent studies have revealed its role in DLBCL (162). IL-16 recruits CD4+ monocytes into the TME, promoting macrophage infiltration, angiogenesis, and upregulation of tumor-promoting cytokines such as IL-6 and IL-10 (162). Notably, IL-16 also suppresses T cell infiltration, collectively facilitating tumor progression (162).

Cytokines and their metabolic effects are not only mechanistic drivers but also valuable prognostic markers. Elevated serum levels of IL-6 and IL-10 are consistently associated with poor prognosis, higher tumor aggressiveness, and shorter survival in patients with DLBCL and FL (163–166). LDH, a key enzyme reflecting cytokine-driven glycolysis, remains a classic independent prognostic factor in both the DLBCL IPI and FL risk models. Moreover, the abundance of immunosuppressive cells such as M2 tumor-associated macrophages, induced by cytokines like IL-10, has been linked to adverse outcomes and higher risk of histologic transformation—particularly in FL (167, 168).

Understanding how specific cytokines drive metabolic programs provides a biological rationale for risk stratification and therapy development. Although agents such as the IL-6R monoclonal antibody tocilizumab have demonstrated potential in reversing cytokine-induced metabolic dependency, challenges remain due to cytokine network redundancy and microenvironmental heterogeneity (169). Future research should focus on identifying dominant cytokine-metabolism axes in different lymphoma subtypes and investigating their downstream metabolites as dynamic biomarkers to inform personalized treatment strategies.

#### 2.2.5 Immune checkpoint molecules and tumor metabolism

The PD-1/PD-L1 axis, a central immune checkpoint pathway, not only suppresses T cell anti-tumor activity but also directly reshapes the metabolic landscape of the TME through metabolic reprogramming (170–172). n B-cell lymphomas, PD-1 activation downregulates the PI3K/Akt/mTOR pathway and its downstream effector MYC, resulting in suppressed glycolysis and enhanced fatty acid β-oxidation (172, 173). This metabolic shift contributes to T cell exhaustion and reinforces PD-1 expression—forming a feedforward loop—while also remodeling the TME to favor immune evasion and tumor progression (174). Interestingly, PD-1/PD-L1 interactions may also activate oncogenic signaling in tumor cells. Dong et al. demonstrated that PD-1 binding to PD-L1 directly activates the AKT/mTOR pathway in DLBCL cells (175),echoing findings from Lastwika et al. in NSCLC, where AKT/mTOR activation upregulates PD-L1 expression to facilitate immune escape (176), These results suggest a positive feedback loop between PD-1/PD-L1 and PI3K/AKT/mTOR signaling that may enhance DLBCL aggressiveness. Hence, combined blockade of PD-1/PD-L1 and AKT/mTOR signaling may represent a promising therapeutic strategy for specific DLBCL subsets.

Emerging checkpoints such as LAG-3 and TIM-3 have attracted attention for their immunomodulatory roles in DLBCL and FL, although their involvement in metabolic regulation remains to be fully elucidated. LAG-3 co-expresses with PD-1 and suppresses T cell function, possibly through similar metabolic mechanisms (177, 178). LAG-3 is also a surface marker of Tregs and may facilitate their suppressive function (179). Notably, Tregs suppress effector T cells through metabolic competition—such as glucose depletion—suggesting that LAG-3 may participate in shaping T cell metabolic reprogramming (180). TIM-3 may impair mitochondrial function and oxidative phosphorylation via the HMGB1/galectin-9 axis, thereby promoting immune suppression and altering TME metabolism (181–183). Co-expression of LAG-3 and TIM-3 with PD-1 may amplify these metabolic effects (184). Preclinical studies indicate that dual blockade of LAG-3 and PD-1 reduces tumor progression and improves anti-tumor T cell responses, underscoring the potential role of metabolic reprogramming as a convergent downstream effect of multi-checkpoint inhibition (185).

However, direct evidence of LAG-3 and TIM-3 regulating metabolism in B-cell lymphomas remains limited. Integrative single-cell metabolomics and immune checkpoint profiling are needed to elucidate whether these molecules contribute to immune escape through metabolic reshaping, and to inform the design of rational combination therapies targeting both immune checkpoints and tumor metabolism.

## 3 Clinical relevance of metabolic parameters

### 3.1 Definition and measurement of tumor metabolic volume and glycolytic activity

TMV and glycolytic activity are critical parameters reflecting the metabolic state of tumors, commonly assessed by positron emission tomography/computed tomography (PET/CT) combined with radiolabeled glucose analogs such as ^18^F-fluorodeoxyglucose (^18^F-FDG) (186, 187). TMV represents the volumetric extent of FDG uptake within tumor tissue, thereby illustrating the spatial distribution of metabolically active tumor cells (188). Glycolytic activity is quantified by standardized uptake values (SUV), indicating the intensity of glucose uptake and metabolism by tumor cells (189). These parameters not only provide a direct visualization of tumor metabolic activity but also enable dynamic assessment of intratumoral heterogeneity. With advancements in imaging technologies, PET/CT-derived metabolic parameters have become indispensable tools for diagnosis, staging, and therapeutic response evaluation in DLBCL and FL (190).

### 3.2 Correlation of metabolic parameters with disease risk and prognosis

The prognostic value of metabolic parameters such as TMV and glycolytic activity in DLBCL and FL has been extensively investigated and closely associates with disease risk and clinical outcomes. In DLBCL, elevated TMV and increased glycolytic activity typically denote a more aggressive disease phenotype and correlate significantly with adverse prognostic factors, including higher IPI scores and advanced disease stages, as well as inferior treatment responses (191). Moreover, enhanced metabolic activity may promote tumor immune evasion by lactate-mediated suppression of T cell function, further accelerating disease progression. Similarly, in the indolent yet potentially transformative FL, elevated metabolic activity, as evidenced by increased FDG uptake, associates with higher histologic grades, greater risk of disease progression and transformation, and shortened PFS (192–195). Metabolic parameters have emerged as robust prognostic indicators. Baseline TMV and total lesion glycolysis (TLG) serve as independent predictors of poor PFS and overall survival (OS) in DLBCL (196, 197), whereas metabolic remission status assessed by interim PET/CT effectively predicts therapeutic response and relapse risk in FL, correlating with long-term survival outcomes (198). These findings underscore the utility of metabolic parameters in risk stratification and individualized treatment decision-making. For instance, DLBCL patients exhibiting high metabolic activity might benefit from intensified therapeutic regimens or agents targeting metabolic pathways, while FL patients with low metabolic activity may be candidates for watchful waiting strategies.

Although metabolic parameters show great prognostic potential, their clinical application still faces challenges. The most critical point is that there are differences in pet/ct scanners, imaging protocols and image analysis software used by different medical institutions, which may lead to the lack of direct comparability of the measured values of SUVmax, TMV, TLG and other parameters (199). This standardization problem limits the wide applicability of these indicators in cross center research and clinical practice to a certain extent. In the future, it is necessary to solve this problem by establishing unified imaging guidelines, calibration standards and automated analysis processes.

## 4 Clinical relevance of immune parameters

### 4.1 Definition and measurement of TIL density and immune checkpoint molecule expression

TILs density and expression of immune checkpoint molecules such as LAG-3 and TIM-3 are key metrics for evaluating the tumor immune microenvironment. TIL density refers to the quantity and spatial distribution of lymphocytes infiltrating tumor tissue—primarily CD8+ and CD4+ T cells—quantified by immunohistochemistry (IHC) or multiplex immunofluorescence techniques. The expression levels of immune checkpoints PD-L1, LAG-3, and TIM-3 are typically assessed by IHC or flow cytometry. PD-L1 expression on tumor cell surfaces binds to PD-1 on T cells, suppressing their activation and function to facilitate immune evasion. Similarly, LAG-3 (lymphocyte activation gene-3) and TIM-3 (T cell immunoglobulin and mucin domain-containing protein-3) are inhibitory receptors on T cells; their overexpression also dampens anti-tumor T cell activity, exacerbating immune escape. The synergistic action of these molecules creates a multifaceted immunosuppressive milieu, impacting the efficacy of immunotherapies.

### 4.2 Association of immune parameters with disease risk and prognosis

As discussed in the previous section (2.2.5), immune checkpoint molecules such as PD-1/PD-L1 can mediate immunosuppression through metabolic reprogramming, and their expression levels also have important prognostic value in clinic. In both DLBCL and FL, TIL density and the expression levels of PD-1/PD-L1 and emerging immune checkpoints LAG-3 and TIM-3 correlate strongly with disease risk and prognosis. In DLBCL, higher TIL density—particularly CD8+ T cell infiltration—is generally associated with favorable outcomes, including prolonged PFS and OS (200–202). However, the presence of immunosuppressive cells such as Tregs and M2 macrophages may attenuate CD8+ T cell anti-tumor efficacy and affect therapeutic responses (112, 202). PD-L1 expression in DLBCL has a dual role: high expression may reflect tumor immune evasion via checkpoint pathways (203), but also predicts enhanced sensitivity to PD-1/PD-L1 blockade therapies (204). Emerging immune checkpoints LAG-3 and TIM-3 are increasingly recognized for their immunoregulatory functions in DLBCL. LAG-3 is highly expressed on exhausted T cells, contributing to T cell dysfunction and immune escape (205), with its expression linked to shorter disease-free survival (DFS) (206). Similarly, TIM-3 expression correlates with T cell exhaustion and portends poorer prognosis (207). Notably, co-expression of PD-1, TIM-3, and LAG-3 is associated with inferior PFS and OS, suggesting that combinatorial blockade of these checkpoints could be a promising therapeutic avenue (208).

The immune microenvironment of FL is typically characterized by profound immunosuppression, where diverse immune cell populations collectively influence disease progression and transformation risk. Elevated CD68+ macrophage counts, diffuse infiltration of FOXP3+ Tregs, and high PD-L1 expression are all linked to shorter time to transformation (209). Remarkably, pre-treatment PD-1 expression in tumor tissue predicts subsequent FL transformation into DLBCL (210). In addition to PD-1, other potential predictive markers have also attracted much attention, such as high-frequency gene mutations (such as EZH2, TP53), the gene map of circulating tumor DNA (ctDNA), and the distribution characteristics of M2 macrophages or specific T cell subsets in the microenvironment (211–214). Integrating multi omics markers may build a more accurate transformation prediction model.LAG-3 and TIM-3 also play critical roles in FL immune escape. The FL microenvironment often features exhausted T cells, with high LAG-3 and TIM-3 expression further amplifying immunosuppression. Increased LAG-3+ TILs associate with disease progression and poor prognosis (215), whereas elevated TIM-3 expression may reduce immunotherapy responsiveness (216). These findings highlight the potential of targeting LAG-3 and TIM-3, particularly in combination with PD-1/PD-L1 inhibitors, to develop novel treatments for FL.

## 5 Conclusion and perspectives

The interaction between metabolism and immune microenvironment opens up a new way for the treatment of DLBCL and FL. Studies have shown that metabolic reprogramming and immunosuppressive microenvironment characteristics are closely related to disease progression, treatment response and prognosis, which provides a theoretical basis for the development of innovative therapies (217, 218). In terms of clinical transformation, targeting key metabolic pathways such as IDHA inhibitors to regulate glycolysis, IDO1 inhibitors to interfere with tryptophan metabolism, and CD73 inhibitors to block adenosine signaling can effectively reverse the immunosuppressive state and enhance the antitumor activity of T cells and NK cells (219–221). In the field of immunotherapy optimization, for the limited efficacy of PD-1/PD-L1 inhibitors, the combination of metabolic regulators such as metformin or novel immune checkpoint inhibitors such as LAG-3/Tim-3 blockers is expected to improve the treatment response (222, 223). At the same time, the strategies of regulating macrophage polarization to promote M1 transformation and inhibiting Treg function by targeting CD39/adenosine axis also showed good application prospects (26, 224). In terms of prognosis evaluation, the integration of metabolic parameters such as TMV and glycolytic activity, as well as immune characteristics such as TILs density and PD-L1/LAG-3 expression can significantly improve the prediction accuracy of existing prognosis models such as IPI and FLIPI (225, 226), while dynamic monitoring technologies such as PET/CT combined with liquid biopsy provide new ideas for early efficacy evaluation and recurrence warning (227, 228).

Looking forward to the future, multi omics integration research including single-cell sequencing, spatial transcriptome and metabonomics will deeply reveal the cell-specific mechanism of metabolism immune interaction, especially focusing on the key drivers of FL to DLBCL transformation (145, 229). In terms of treatment strategy development, it is very important to design a joint scheme targeting the dual pathways of metabolism and immunity, such as PHGDH inhibitor combined with PD-1 inhibitor, and verify its efficacy through clinical trials. The combination of strategies targeting the metabolism immune axis and emerging immunotherapies (such as CAR-T cells and bispecific antibodies) has great potential (92, 230). For example, the use of IDHA inhibitors or A2AR antagonists to improve the immunosuppressive status of TME may reverse the depletion of CAR-T cells *in vivo* and enhance their persistence and anti-tumor efficacy (230, 231). Similarly, during bispecific antibody therapy, simultaneous intervention of adenosine or tryptophan metabolic pathways is expected to relieve the inhibition of endogenous T cells and produce synergistic anti-tumor effects (92). These joint strategies will become an important direction of clinical transformation research in the next step. At the same time, microenvironment remodeling strategies such as regulating cytokines such as IL-6/IL-10 or intervening in nutritional competition may significantly enhance treatment sensitivity (166, 232, 233). In terms of technological innovation, the development of non-invasive monitoring technologies such as imageomics virtual biopsy, and the establishment of PDX model and organ like platform will greatly promote the process of drug research and development and clinical transformation. Finally, an individualized treatment system based on patient specific metabolic and immune characteristics such as TME typing, such as the design of IDHA inhibitors combined with immunotherapy for patients with high lactic acid microenvironment, will promote lymphoma treatment into a new era of precision medicine.
